# The Type III Secretion System-Related CPn0809 from *Chlamydia pneumoniae*

**DOI:** 10.1371/journal.pone.0148509

**Published:** 2016-02-19

**Authors:** Astrid C. Engel, Frauke Herbst, Anne Kerres, Jan N. Galle, Johannes H. Hegemann

**Affiliations:** Lehrstuhl für Funktionelle Genomforschung der Mikroorganismen, Heinrich-Heine-Universität, Düsseldorf, Germany; University of California, San Francisco, University of California, Berkeley, and the Children's Hospital Oakland Research Institute, UNITED STATES

## Abstract

*Chlamydia pneumoniae* is an intracellular Gram-negative bacterium that possesses a type III secretion system (T3SS), which enables the pathogen to deliver, in a single step, effector proteins for modulation of host-cell functions into the human host cell cytosol to establish a unique intracellular niche for replication. The translocon proteins located at the top of the T3SS needle filament are essential for its function, as they form pores in the host-cell membrane. Interestingly, unlike other Gram-negative bacteria, *C*. *pneumoniae* has two putative translocon operons, named LcrH_1 and LcrH_2. However, little is known about chlamydial translocon proteins. In this study, we analyzed CPn0809, one of the putative hydrophobic translocators encoded by the LcrH_1 operon, and identified an ‘SseC-like family’ domain characteristic of T3S translocators. Using bright-field and confocal microscopy, we found that CPn0809 is associated with EBs during early and very late phases of a *C*. *pneumoniae* infection. Furthermore, CPn0809 forms oligomers, and interacts with the T3SS chaperone LcrH_1, via its N-terminal segment. Moreover, expression of full-length CPn0809 in the heterologous host *Escherichia coli* causes a grave cytotoxic effect that leads to cell death. Taken together, our data indicate that CPn0809 likely represents one of the translocon proteins of the *C*. *pneumoniae* T3SS, and possibly plays a role in the translocation of effector proteins in the early stages of infection.

## Introduction

*Chlamydia pneumoniae* is an obligate intracellular Gram-negative pathogen that causes a wide range of pulmonary diseases. Because these are often mild and atypical in character, it is thought that the bacterium’s contribution to the incidence of respiratory illness is significantly underestimated [1]. In addition, *C*. *pneumoniae* can induce persistent infections and has been implicated as a subsidiary factor in other severe respiratory diseases, including asthma, chronic obstructive pulmonary disease (COPD) and lung cancer, and is suspected of playing a role in other pathologies such as atherosclerosis, Alzheimer’s disease and multiple sclerosis [2–4]. Like all *Chlamydiae*, *C*. *pneumoniae* is an obligate intracellular parasite with a unique biphasic life cycle, alternating between a metabolically inert infectious form called an elementary body (EB), which is adapted to survive in the hostile extracellular environment, and an intracellular form called the reticulate body (RB) that replicates by binary fission [5, 6]. The intracellular life cycle of *Chlamydia* depends on the eukaryotic host cell and is initiated by the binding of EBs to the cell surface. The EB is subsequently internalized into a membrane-bound vesicle called an inclusion, in which differentiation and replication of RBs occurs. The inclusion membrane is heavily modified by the bacteria, equipping it for nutrient acquisition and as an intracellular niche for the replication of RBs [7]. After several rounds of replication, the RBs re-differentiate asynchronously back into EBs. Between approximately 48 and 72 h post infection the EBs exit the host cell via lysis or extrusion to invade new cells [5, 8].

During invasion and the establishment and maintenance of the intracellular niche, *C*. *pneumoniae* interacts with its eukaryotic host cell via secreted effector proteins. Like other Gram-negative pathogenic bacteria, such as *Yersinia*, *Salmonella*, *Shigella* and pathogenic *E*. *coli*, the *Chlamydiae* utilize Type III secretion systems (T3SS) to export effector proteins [9, 10]. The T3SS is a syringe-like nanomachine composed of 20 to 25 proteins, which enables the bacterial cell to translocate proteins in a single step across its own inner and outer membranes and through the plasma membrane of a targeted host cell or, in the case of *Chlamydiae*, into the inclusion membrane [11]. The structure that penetrates the plasma membrane of the host cell is called the needle-tip complex, or translocon, and is composed of three proteins [12]. The needle-tip protein or hydrophilic translocator belongs to the LcrV family of proteins, while the two hydrophobic translocators are members of the YopB and YopD families of translocon proteins and are often referred to as the major and minor translocator, respectively. The translocon proteins are themselves secreted via the T3S machinery following contact of the needle with the targeted cell, and are assembled to form a pore in the plasma membrane [12, 13].

Chaperones that bind T3S substrates and maintain them in a secretion-competent state are crucial for the correct function of T3S systems, and might also be involved in determining the sequence of secretion of their substrates [11, 14]. T3SS-associated chaperones are divided into three classes, with the class II chaperones binding to one or both hydrophobic translocators of the T3SS. An archetypal class II chaperone is LcrH/SycD from the well characterized *Yersinia* T3SS, which binds to the hydrophobic translocators and prevents premature folding and homo- or hetero-oligomerization of their substrates in the cytosol of the bacterial cell [11].

**Fig 1:**
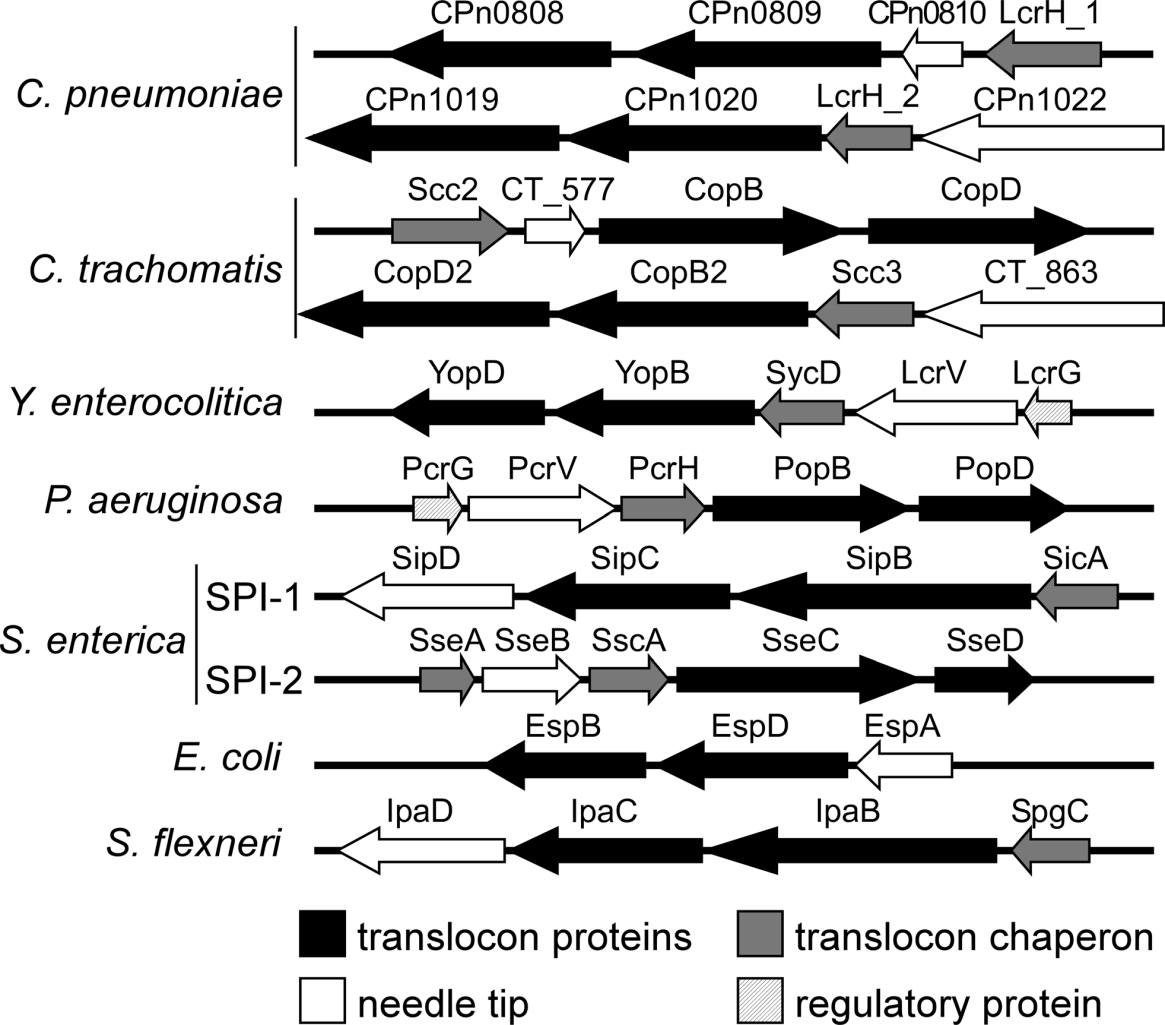
Putative elements of the T3SS encoded by the *C*. *pneumoniae* LcrH_1/2 operon, identified by comparison with those specified by comparable operons in *C*. *trachomatis* and other bacteria. CPn0809 forms part of a T3SS translocon operon structure typical of those found in a variety of pathogenic bacteria. Both *C*. *pneumoniae* and *C*. *trachomatis* harbor two such operons. The organization of T3SS translocon operons is highly conserved among pathogenic Gram-negative bacteria, and several well characterized archetypal translocon operons were chosen for comparison. Putative protein functions are indicated by the color code. The arrows indicate the orientation of gene transcription.

Two putative T3SS class II chaperones were previously identified in *C*. *pneumoniae* by their sequence homology to the *Yersinia* class II chaperone LcrH, and were named LcrH_1 and LcrH_2 [15]. Typically, class II chaperone-coding genes localize next to genes encoding the hydrophobic translocons and the needle-tip proteins, and are expressed from one operon [12, 13]. LcrH_1 is expressed together with CPn0809, CPn0808 and CPn0810, while LcrH_2 is co-expressed with the CPn1019, CPn1020 and CPn1022 proteins (Fig 1) [16]. Interestingly, the two translocon operons are expressed at different stages of infection. The proteins of the LcrH_1 operon are expressed as “tardy” proteins, suggesting that they are stored in the EBs for the next round of infection, while proteins of the LcrH_2 operon are expressed as “mid” class proteins, supporting the idea that both operons function at distinct phases of the developmental cycle [16, 17]. The two putative translocon protein sets are poorly characterized. Based on their hydrophobicity profiles, they can be grouped into pairs of hydrophobic translocators (CPn0808 and CPn0809 in the first operon; CPn1019 and CPn1020 in the second operon) and the corresponding hydrophilic needle-tip proteins (CPn0810 and CPn1022, respectively) (Fig 1). Very recently CPn0808 was found to be essential for successful infection by *C*. *pneumoniae* [18]. So far, CPn0809 has not been characterized in detail. An early study localized the protein as a secreted effector in the host-cell cytosol during infection [19]. Recently, human interaction partner candidates for CPn0809 were identified in a yeast two-hybrid assay [20]. These data suggest that CPn0809 is a soluble effector protein which interacts with host proteins in the host cytosol, in contrast to early bioinformatics analyses suggesting CPn0808 and CPn0809 to be translocator proteins [15].

In this study, we characterize CPn0809 bioinformatically and by determining its expression pattern and subcellular localization during the chlamydial life cycle. We found that CPn0809 co-localizes with chlamydial EBs at the target-cell membrane and during the first several hours of infection, and again becomes associated with progeny EBs late in infection. Furthermore, using genetic and biochemical assays, we show that CPn0809 interacts with itself and with the class II chaperone LcrH_1. These results, and the strong growth inhibition phenotype observed upon expression in *E*. *coli*, are consistent with the assumption that CPn0809 is the major translocator of a *C*. *pneumoniae* T3SS translocon, with a function during target-cell attachment and invasion.

## Methods and Materials

### Ethic statement

For the immunization, the rabbits were handled in strict accordance to the good animal practice as defined by the Belgian national animal welfare regulations, and all animal work was approved by the ethics committee of the Centre d'Economie Rurale (CER Groupe, Marloie, Belgium). All rabbit handlings were performed at Eurogentec SA, Seraing, Belgium, under permit number LA 1800104.

Please find hereafter regulations followed by Eurogentec animal facility regarding animal care, housing and transportation: Welfare Legislation for laboratory animals c 2010/63/EU Animal Transportation c 01/2005/EU Pharmacy c RD29/06/199 c RD23/05/2000 c RD19/12/2002 Others c Agreement STE123 c Scientific procedures: Animals Act 1986 c 2004/21/EU Identification of ovine and caprine species.

### Culture conditions and organisms

*Escherichia coli* strain XL1-Blue (Stratagene) was used for protein expression and plasmid amplification. *Chlamydia pneumoniae* GiD [21] was grown in HEp-2 cells in the presence of 1.2 μg/ml cycloheximide as described previously [22]. Chlamydial EBs were purified using a 30% gastrographin gradient (Bayer).

The *Saccharomyces cerevisiae* strain CEN.PK2 *MAT***a**/α *leu*2-3_112/*leu*2-3_112 *ura*3-52/*ura*3-52 *trp*1-289/*trp*1-289 *his*3-D1/*his3*-D1 *MAL*2-8C/ *MAL*2-8C *SUC*2/*SUC*2 [23] was used for cloning, and strain AH109 (Matchmaker^®^Gold Clontech) *MAT***a** *trp1-901 leu2-3_112 ura3-52 his3-200 gal4Δ gal80Δ LYS2*::*GAL1*_*UAS*_*-GAL1*_*TATA*_*-His3 GAL2*_*UAS*_*-GAL2*_*TATA*_*-Ade2 URA3*::*MEL1*_*UAS*_*-MEL1*_*TATA*_ *LacZ MEL1* for Y2H experiments. Both were routinely grown either on YPD or on plasmid-selective synthetic medium containing 2% glucose (SD) [24].

The epithelial larynx carcinoma cell line HEp-2 (ATCC^®^ #: CCL-23™) was cultivated in Dulbecco’s modified Eagle medium (DMEM) GlutaMax™ (Life Technologies) supplemented with 10% fetal calf serum (FCS), vitamins, nonessential amino acids, amphothericin B (2.5 μg/ml) and gentamicin (50 μg/ml).

### Bioinformatic analyses

Protein sequences were aligned using BLASTp (http://blast.ncbi.nlm.nih.gov/Blast.cgi?PROGRAM=blastp&PAGE_TYPE=BlastSearch&LINK_LOC=blasthome) with default parameters [25]. Protein family comparisons were performed with the full-length proteins using Pfam (http://pfam.xfam.org/) [26]. TMHMM 2.0 (http://www.cbs.dtu.dk/services/TMHMM/) and Phobius (http://phobius.sbc.su.se/) were used to locate hydrophobic domains and Marcoil (http://bcf.isb-sib.ch/webmarcoil/webmarcoilC1.html) was used to detect coiled-coil domains in full-length proteins [27–29].

### DNA manipulation and plasmid constructions

Recombinant plasmids were generated by homologous recombination in *S*. *cerevisiae*. DNA fragments were amplified by PCR from *C*. *pneumoniae* GiD genomic DNA using oligonucleotides with 40 nucleotides of homology to the targeted vector and 20 nucleotides of homology to the inserted DNA. All PCR products were integrated into linearized plasmids (Tables1 and 2). The recombinant plasmids were rescued as described previously [30].

**Table 1:** Plasmids used in this study.

Plasmid	Properties	Source or reference
**pGBKT7**	Yeast two-hybrid (Y2H) vector containing the *GAL4* DNA-binding domain	Matchmaker^®^Gold Clontech
**pGADT7**	Yeast two-hybrid (Y2H) vector containing the *GAL4* activation domain	Matchmaker^®^Gold Clontech
**pEB1**	Altered pGADT7 vector, coding for a additional His_6_ tag-sequence downstream of the multiple cloning site	[31]
**pKM32**	*E*.* coli* expression vector for creation of N-terminal His_6_-containing fusion proteins	[32]
**pFT8**	*E*.* coli* expression vector for creation of N-terminal GST- and C-terminal His_6_-containing fusion proteins	[33]
**pFT34**	*E*.* coli* expression vector for CPn0473 with a C-terminal His_10_-tag	Tim Fechtner and Johannes H. Hegemanan, unpubl.

**Table 2:** Oligonucleotides used for amplification of DNA for insertion into plasmids.

Insert DNA and plasmid	Forward and reverse primers used for PCR amplification of insert DNA
*cpn0809*N (aa 1–253) in pGBDT7	5’GGAGCAGAAGCTGATCTCAGAGGAGGACCTGCATATGGCC**ATGTCTATTTCATCTTCTTCAGGA** 3’ and 5’CAAGGGGTTATGCTAGTTATGCGGCCGCTGCAGGTCGACG**TTAGACAGTATCCATTGTTCCTTC**-3’
*lcrH_1 (cpn0811)* in pGADT7	5’GGAGTACCCATACGACGTACCAGATTACGCTCATATGGCC**ATGAGCAAGCCCTCTCCTCG** 3’ and 5’ACGATTCATCTGCAGCTCGAGCTCGATGGATCCCGTATCG**CTAACGTTTCTTTCCGCTTTTC** 3’
*lcrH_1 (cpn0811)* in pKM32	5’GAAATTAACTATGAGAGGATCTCACCATCACCATCACCAT**ATGAGCAAGCCCTCTCCTCG** 3’ and 5’TAATTAAGCTTGGCTGCAGGTCGACCCGGGGTACCGAGC**CTAACGTTTCTTTCCGCTTTTC** 3’
*cpn0809*N (aa 1–253) in pEB1	5’CGCCGCCATGGAGTACCCATACGACGTACCAGATTACGCT**ATGTCTATTTCATCTTCTTC** 3’ and 5’GGTTTTTCAGTATCTACGATTCATGGTGATGGTGATGGTG**GACAGTATCCATTGTTCCTT** 3’
*cpn0809* (aa 1–488) in pKM32	5’CCATCACCATCACCATACGGATCCGCATGCGAGCTCGGTA**ATGTCTATTTCATCTTCTTCAG** 3’ and 5’CAACAGGAGTCCAAGCTCAGCTAATTAAGCTTGGCTGCAG**TTATGCTGCGCCAGCGA** 3’
*cpn0809*N (aa 1–253) in pFT8	5’AGGTCGTCTGGAAGTTCTGTTCCAGGGGCCCCTGGGATCC**ATGTCTATTTCATCTTCTTC** 3’ and 5’GAGGCAGATCGTCAGTCAGTCAATGGTGATGGTGATGGTG**GACAGTATCCATTGTTCCTTC** 3’
*cpn0809*N (aa 1–253) in pKM32	5’AACTATGAGAGGATCTCACCATCACCATCACCATACGGA**ATGTCTATTTCATCTTCTTC** 3’ and 5’TTGGCTGCAGGTCGACCCGGGGTACCGAGCTCGCATGCGG**TTAGACAGTATCCATTGTTC** 3’
*cpn0809*C (aa 406–488) in pKM32	5’AGGTCGTCTGGAAGTTCTGTTCCAGGGGCCCCTGGGATCC**GGTAAAGGGATTATGCAAATG** 3’ and 5’GAGGCAGATCGTCAGTCAGTCAATGGTGATGGTGATGGTG**TGCTGCGCCAGCGATGGCTC** 3’

Bold letters represent the 20-nucleotide homology to the target gene

All constructs were verified by sequencing (GATC, Konstanz, Germany).

### Protein expression and purification

For expression of recombinant proteins, *E*. *coli* cells harboring the plasmid construct pAF75 (GST-CPn0809N-His_6_), pAF94 (His_6_-CPn0809N), pAE15 (His_6_-LcrH_1), pFT8 (His_6_-GST) or pFT34 (CPn0473- His_10_) were grown in lysogeny broth (LB) medium containing ampicillin (50 mg/l) and harvested 4 h after induction with 1 mM IPTG. Lysis of GST-expressing *E*. *coli* cells was performed under native conditions in phosphate-buffered saline (PBS; 137 mM NaCl, 2.7 mM KCl, 10 mM Na_2_HPO_4_, 1.8 mM KH_2_PO_4_ with 1 mM phenylmethanesulfonylfluoride (PMSF), 0.5 mg/ml lysozyme, 1% Triton X-100, 1 mM dithiothreitol (DTT) and protease inhibitor cocktail (Roche)) overnight at 4°C. The lysate was cleared by centrifugation at 18 400 x g and 4°C for 20 min. Purification of the protein was performed using glutathione-agarose (Sigma-Aldrich) at 4°C. Reduced glutathione was removed by dialysis against PBS at 4°C overnight. For isolation of the His_6_-constructs, lysis of *E*. *coli* cells was performed under denaturing conditions in 8 M urea, 0.1 M NaH_2_PO_4_, 10 mM Tris-HCl (pH 8.0) overnight at room temperature. The lysate was cleared by centrifugation as described above. Purification of the protein was performed on Ni-NTA-agarose (Qiagen) at room temperature. Imidazole was removed and renaturation was performed by dialysis against PBS at 4°C. SDS-PAGE and immunoblot analysis were performed as described previously [34]. Purified protein was probed by immunoblot analysis with anti-His antibody (Qiagen), followed by anti-mouse antibody conjugated with alkaline phosphatase (Promega).

### Antibody generation and purification

For generation of a polyclonal anti-CPn0809 antibody, purified recombinant His_6_-CPn0809 (aa 1–253) protein was used to immunize two rabbits. Immunization was performed first with purified protein cut from a SDS gel and subsequently with native soluble protein. Immunizations were carried out by Eurogentec (Belgium).

The polyclonal anti-CPn0809 antibody was antigen-purified against GST-CPn0809N-His_6_ protein coupled to NHS-activated Sepharose (GE Healthcare Life Sciences) following the manufacturer’s instructions and a standard protocol [35].

### Yeast-2-Hybrid (Y2H) analyses

Two-hybrid analyses were performed using the Matchmaker™ Gold System (Clontech). Combinations of plasmids were transformed into *S*. *cerevisiae* strain AH109, and interaction was tested by serial dilution patch tests on selective medium (Leu^-^, Trp^-^; growth control) and low-stringency medium (Leu^-^, Trp^-^, His^-^). Expression of the constructs was monitored by immunoblot analyses using either an anti-HA antibody (Santa Cruz) or an anti-His antibody (Qiagen). As a positive control the plasmids pGBKT7-53 (human protein p53) and pGADT7-T (large T-antigen of SV40) and as the negative control pGBKT7-Lam (lamin C) and pGADT7-T were co-transformed into yeast cells.

### Far Western Analysis

Recombinant proteins were expressed and lysed under native conditions. After centrifugation, a 50-μg aliquot of protein solution was fractionated via SDS-PAGE and transferred to a PVDF membrane. The immobilized prey proteins were renatured gradually by incubating the membrane successively for 30 min each in 6 M, 3 M, 1 M guanidine HCl at room temperature, 0.1 M guanidine at 4°C and finally without guanidine at 4°C overnight. Sulfo-NHS-Biotin (Pierce) was added to the bait protein with a 20-fold molar excess. The reaction was incubated on ice for two hours and stopped by adding Tris-HCl (pH 8.0) to a final concentration of 50 mM. The biotinylated protein was dialyzed overnight against PBS to remove excess biotin. Biotinylated bait protein (2 mg/ml) was added to the renatured, immobilized prey proteins for 2 h at room temperature. The prey proteins were visualized with anti-His antibody (Qiagen), followed by anti-mouse antibody conjugated with alkaline phosphatase (Promega), and the complex formed between bait and prey was detected with alkaline phosphatase-conjugated streptavidin.

### Immunoblot analysis of CPn0809 during the *C*. *pneumoniae* life cycle

HEp-2 cells grown in 25 cm² cell culture flasks were infected with *C*. *pneumoniae* GiD (MOI 10). At each time point chosen, the cell culture medium was discarded and the cells were harvested with a cell scraper, resuspended in 10 ml of Hanks’ Balanced Salt Solution (HBSS) and sedimented for 20 min at 2 540 x g at 4°C. The supernatant was discarded and the cell pellet lysed in 300 μl lysis buffer (2% SDS, 2% Sarkosyl, 1% IGEPAL CA-630, 1% Triton X-100, 140 mM NaCl, 20 mM Tris-HCl pH 7.5, 2 mM EDTA, 1 mM Na_2_VO_4_).

Equal volumes of lysate were analyzed for each time point using antibodies against β-actin (Sigma-Aldrich), chlamydial DnaK (kindly provided by the Christiansen laboratory [36]) and CPn0809, respectively. Secondary antibodies conjugated with alkaline phosphatase (Promega) were used for visualization.

### Detergent treatment

Gradient-purified *C*. *pneumoniae* GiD EBs were treated with detergents to determine if specific proteins can be solubilized from the chlamydial surface. Aliquots (75μl) of purified EBs (~10^9^ IFU/ml) were pelleted for 30 min at 21 800 x g and 4°C. The pellet was resuspended either in 150 μl PBS with 1% Triton X-100, PBS with 2% Sarkosyl or PBS alone, and incubated for 1 h at 37°C. Subsequently, the sample was centrifuged for 1 h at 4°C and 100 000 x g. Pellet and supernatant were analyzed on immunoblots with antibodies against MOMP [37], GroEL1 [30], EF-Tu (Stallmann, unpublished) or CPn0809.

### Microscopy

HEp-2 cells were grown on glass cover slips (12 mm diameter) and infected for different periods of time. Cells were then fixed with cold (-20°C) methanol for 5 min.

For indirect immunofluorescence analyses, antibodies against IncA (kindly provided by the Zhong laboratory [38]), LPS (Bio-Rad), DnaK (see above) and CPn0809 were visualized with specific secondary antibodies conjugated to Alexa-488 or Alexa-594 (Life Technologies). Wheat germ agglutinin (WGA) (Life Technologies) directly conjugated to Alexa-594 was used to stain the plasma membrane of the host cell. DNA was visualized using 4',6-diamidino-2-phenylindole (DAPI).

Vital staining of *E*. *coli* cells with propidium iodide was performed as follows: *E*. *coli* cells harboring plasmid constructs for expression of His_6_-CPn0809 (pAE41), His_6_-CPn0811 (pAE15), or bearing the empty vector (pKM32), were grown in liquid LB medium with ampicillin (50 mg/l). Cells from an overnight culture were used to inoculate 50 ml of ampicillin-containing (50 mg/l) LB medium to an OD_600_ of 0.05. Protein expression was induced with 1 mM IPTG at an OD_600_ of 0.6 for 2 h at 37°C. *E*. *coli* cells equivalent to 1 OD_600_ unit were harvested and washed twice with 1 ml 10 mM Tris-HCl pH 7.5 and centrifuged for 3 min at 7 400 x g. The supernatant was discarded and cells were stained for 3 min with propidium iodide (5 μg/ml) in 10 mM Tris-HCl pH 7.5. Propidium iodide was removed by washing the cells twice with 1 ml 10 mM Tris-HCl pH 7.5. Immediately after staining, the cells were analyzed by fluorescence microscopy.

Images were acquired using either a Zeiss fluorescence microscope Axioskop 50 equipped with a 12-bit monochrome charge-coupled device (CCD) camera (CHROMOPHOR Analysetechnik GmbH, Germany) or a Nikon confocal microscope C2 and Nikon NIS-Elements AR imaging software (Nikon). All images were processed using Canvas 14 (ACD Systems of America, Inc.).

### Antibody pre-adsorption assay

To check the antibody specificity antigen-purified polyclonal anti-CPn0809 antibody was pre-adsorbed against immobilized recombinant GST-CPn0809N-His_6_ protein. Green fluorescent latex beads (1 x 10^9^, ø 1.1 μm, Polyscience) were coupled with 100 μl of the recombinant protein dissolved at 400 μg/ml in coupling buffer (0.2 M NaHCO_3_, 0.5 M NaCl2, pH 8.6) as described before [39] or just blocked by incubation with 500 μl BSA (40 mg/ml) as a control. Anti-CPn0809 antibody (100 μl) was incubated with protein-coated beads overnight at 4°C under agitation. The suspension was centrifuged for 5 min at 13 900 x g and the supernatant was used for immunofluorescence staining of methanol-fixed, infected HEp-2 cells. As a control, beads were resuspended in PBS and incubated with a secondary Alexa-594-conjugated antibody to show adsorption of anti-CPn0809 antibody.

## Results

### CPn0809 possesses an ‘SseC-like family’ domain characteristic of T3S translocators

**Fig 2:**
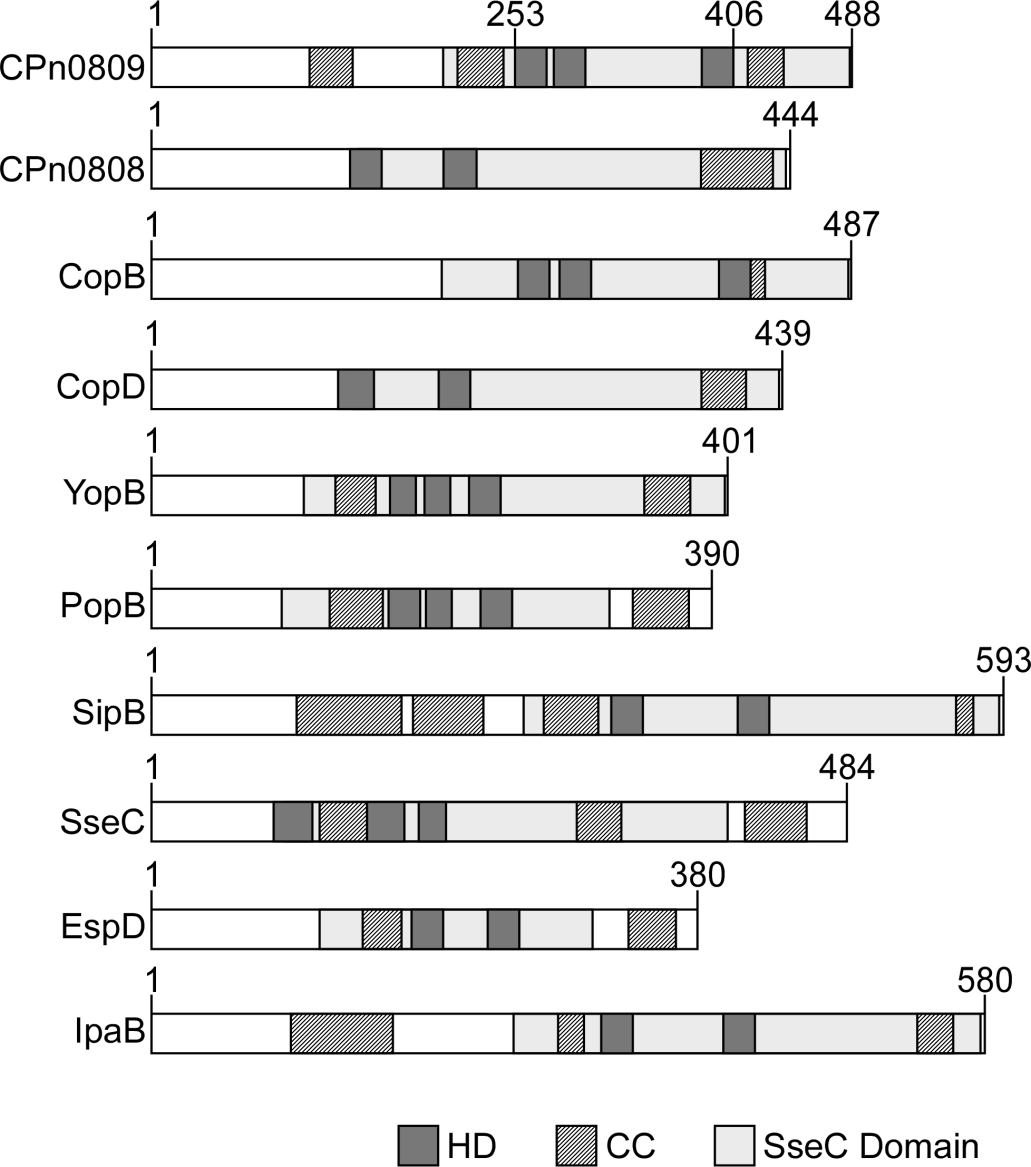
Structural comparison of translocon proteins harboring an ‘SseC-like family’ domain. Comparison of *C*. *pneumoniae* CPn0809/CPn0808 and the *C*. *trachomatis* CT_578 (CopB)/CT_579 (CopD) with translocon proteins containing an ‘SseC-like family’ domain from other Gram-negative bacteria (see Fig 1 for species). Numbers indicate amino acid positions. The distribution of predicted ‘SseC-like family’, hydrophobic (HD), and coiled-coil (CC) domains within CPn0809 and other major translocon proteins is shown schematically. The SseC domain was identified by Pfam search. HD domains were predicted with the prediction program Phobius, and TMHMM 2.0. CC domains were predicted by Marcoil. All bioinformatic analyses were performed on the full-length protein sequences.

As shown in Fig 1, the translocon operons of different bacterial species generally encode two hydrophobic and one hydrophilic translocon proteins, which are co-expressed together with a class II chaperone and, in some cases, other regulatory proteins. In *C*. *pneumoniae* CPn0809 is co-expressed with LcrH_1 and with CPn0808 and CPn0810 from one operon [16]. Bioinformatic analysis suggests that CPn0809 harbors three hydrophobic domains (HD) in its C-terminal half (aa 253–488), which are flanked by two coiled-coil domains (CC), while a third CC domain is located in the N-terminal segment (Fig 2). Thus CPn0809 exhibits structural similarities to members of the YopB family of translocators, also known as major hydrophobic translocators [12, 15]. In addition, an ‘SseC-like family’ domain was identified in the C-terminal portion of CPn0809 (aa 203–487). This domain is found in all major hydrophobic translocators of other T3SS-expressing bacteria examined (Fig 2). In general the SseC domains of the translocon proteins display common features, including two or more hydrophobic domains (HD) combined with one or more coiled-coil domains (CC). In most cases, the SseC and hydrophobic domains encompass the C-terminal end of the protein (see Fig 2).

CPn0809 and its *C*. *trachomatis* homolog CT_578/CopB were compared to other SseC domain-containing translocon proteins from other species. In other bacteria only the major hydrophobic translocator contains a predicted SseC domain. Intriguingly, an SseC domain is predicted in both hydrophobic translocators encoded by the *C*. *pneumoniae* LcrH_1 operon but not in either of those of the LcrH_2 operon (Fig 2). A similar situation is found for the hydrophobic translocators of *C*. *trachomatis*. Taken together, these findings reveal differences between chlamydia and other Gram-negative bacteria. Thus the bioinformatic data suggest that CPn0809 might function as a major translocon protein, very much like other polypeptides harboring an ‘SseC-like family’ domain.

### CPn0809 is associated with EBs

**Fig 3:**
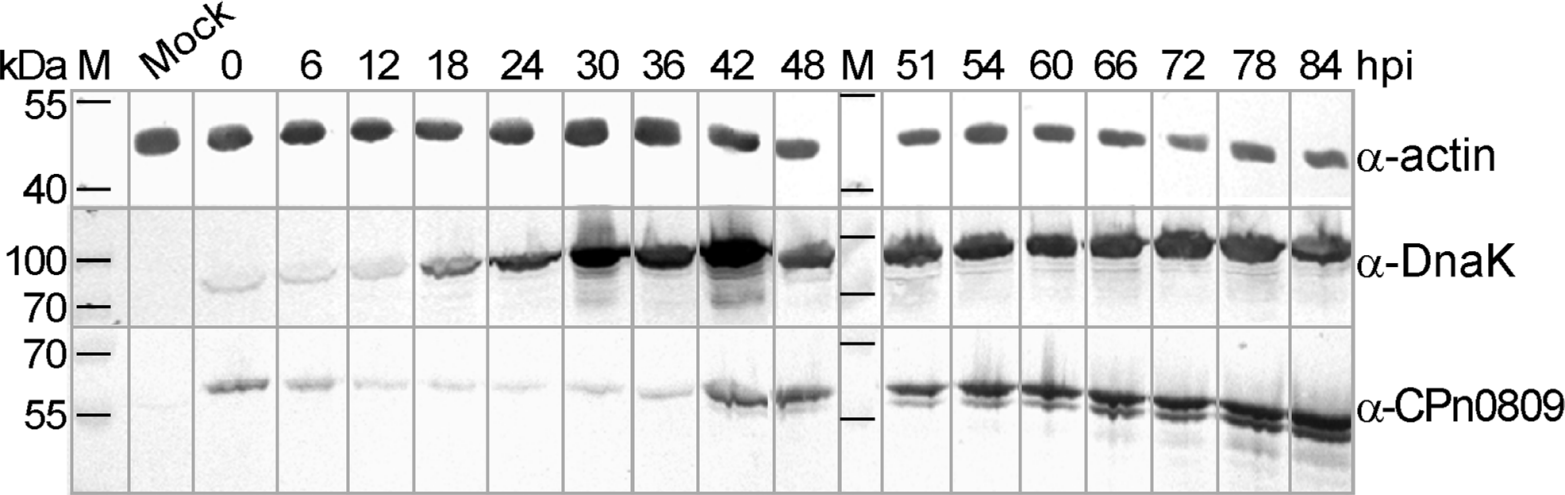
Expression of CPn0809 during the *C*. *pneumoniae* developmental cycle. Confluent HEp-2 cell cultures were either mock-infected or infected with *C*. *pneumoniae* GiD (MOI 10) and harvested at various times post infection (hpi) as indicated at the top of the Fig. Cells were lysed and lysates were probed with a specific polyclonal anti-CPn0809 antibody (50 kDa). Antibodies specific for the chlamydial chaperone DnaK (72 kDa) and human β-actin (42 kDa) were used to check equivalence of loading. Secondary antibodies conjugated with alkaline phosphatase were used for visualization. M = protein size marker.

First we characterized the expression of CPn0809 during the *C*. *pneumoniae* infection cycle by immunoblot analysis. Using an antibody directed against the CPn0809 N-terminal half (aa 1–253) we were able to detect CPn0809 throughout the entire developmental cycle (Fig 3). Significant amounts of CPn0809 were found to be present at early time points in infection (0–6 hpi). Subsequently the signal intensity decreased, reaching a minimum at 24 hpi. At this point most bacteria begin to replicate, as indicated by the increased signal for the DnaK protein. From 42 hpi on, CPn0809 levels show a marked rise. During this phase of the infection cycle, the *C*. *pneumoniae* RBs re-differentiate asynchronously into EBs [5].

**Fig 4:**
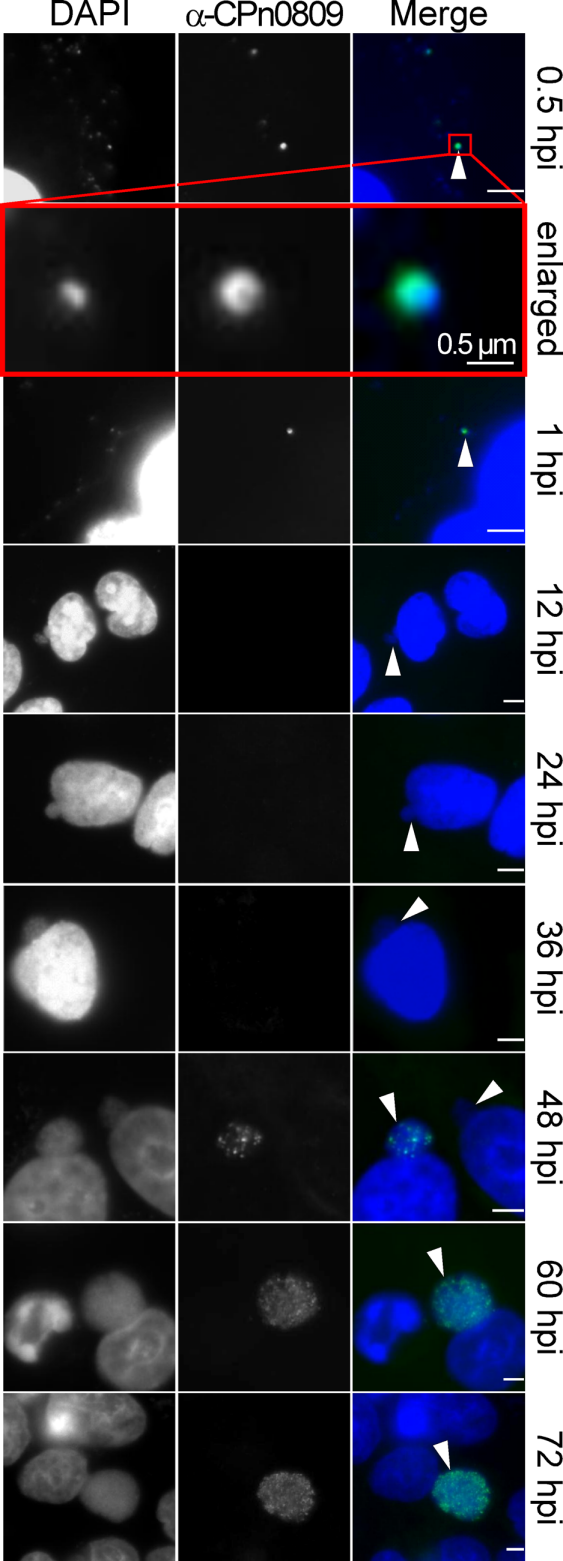
CPn0809 is an EB-associated protein. HEp-2 cells grown on glass cover slips (12 mm diameter) were incubated at 4°C for 10 min and then infected with *C*. *pneumoniae* GiD (MOI 0.5) by centrifugation of an EB suspension at 1 560 x g at 4°C for 10 min. The culture medium was then replaced by pre-warmed fresh medium, and cells were shifted to 37°C. At the indicated time points post infection (hpi) cells were fixed and stained with an anti-CPn0809 antibody and an Alexa-488-conjugated secondary antibody (green). DNA was visualized by staining with DAPI (blue). Images were acquired by epifluorescence microscopy. The marked area in the 0.5-hpi picture is shown enlarged in the demarcated inset. CPn0809-positive particles (0.5–1 hpi) and CPn0809-positive and -negative inclusions (12–72 hpi) are indicated by the arrowheads in the merged images. Scale bar = 5 μm, unless indicated otherwise.

Before proceeding to determine the subcellular localization of CPn0809 during the developmental cycle, we analyzed the specificity of our CPn0809 antibody. After incubation of anti-CPn0809 antibodies with beads coated with the recombinant CPn0809N (aa 1–253), the pre-adsorbed serum failed to recognize CPn0809 during infection, as expected (S1 Fig). We therefore concluded that the punctate CPn0809 signals within the inclusion are specific. At early time points during infection, CPn0809 signals that co-localized with the bacterial DNA were observed (Fig 4). However, due to the asynchronous *C*. *pneumoniae* infection cycle, at 0.5 hpi only 26% (+/- 11%) of the DNA particles were associated with a CPn0809 signal, and this had not changed significantly by 1 hpi. In contrast, between 12 and 36 hpi no signals were obtained. At 48 hpi scattered punctate staining was again observed within the inclusion, although at this point larger inclusions with, and smaller inclusions without CPn0809 signals (arrowhead) could be detected in the same cell. The latter observation probably reflects the asynchronous nature of development at this stage, as the number of CPn0809 signals increased with time until most inclusions seemed to be filled with the protein. At 60 hpi and 72 hpi almost all chlamydial particles within all inclusions exhibited CPn0809 signals.

In contrast to previous reports, we found no evidence that CPn0809 exits the inclusion. The expression and localization pattern indicate that CPn0809 is produced in the second half of the infection cycle and is stored in EBs, suggesting that the protein could be required for the late phase of infection and/or in the early stages of the next round of infection.

### CPn0809 is detectable on the host-cell membrane early in infection

**Fig 5:**
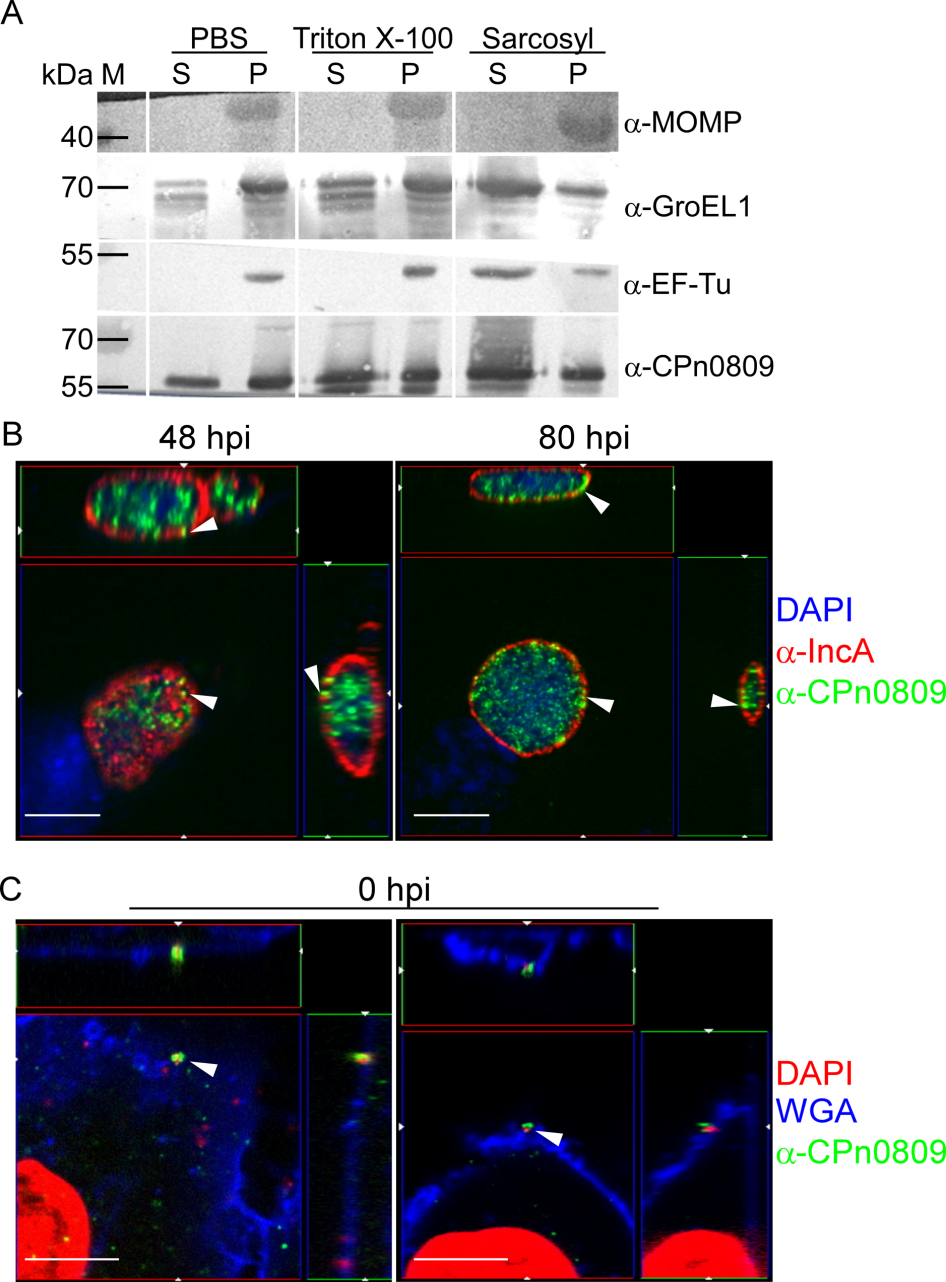
CPn0809 is readily accessible on infectious EBs and is detectable both at very early and at late stages of infection. **A.** Purified EBs where either treated in PBS with or without the detergents Triton X-100 (1%) or Sarkosyl (2%). After ultracentrifugation, pellet (P) and supernatant (S) were analyzed separately by immunoblotting using antibodies directed against the major outer membrane protein (MOMP; 42 kDa), the chlamydial chaperone GroEL1 (58 kDa), the elongation factor EF-Tu (43 kDa) or CPn0809 (50 kDa). The resulting complexes were visualized using specific secondary antibodies conjugated with alkaline phosphatase. **B.** Confocal microscopy of HEp-2 cells infected with *C*. *pneumoniae* (MOI 1) for 48 h or 80 h. Infected cells were stained with the anti-CPn0809 antibody and an Alexa-488-conjugated secondary antibody (green), while the inclusion membrane was stained with a monoclonal mouse anti-IncA antibody and an Alexa-594-conjugated secondary antibody (red). DNA was visualized with DAPI (blue). Arrowheads mark apparent gaps in the IncA distribution that are associated with an adjacent CPn0809 signal. Scale bar, 5 μm. **C.** Pre-cooled HEp-2 cells were infected with *C*. *pneumoniae* GiD at 4°C for 10 min under centrifugation. Immediately after the addition of pre-warmed medium, cells were fixed (0 hpi) and stained with an anti-CPn0809 antibody and an Alexa-488-conjugated secondary antibody (green). Membrane structures were stained with wheat-germ agglutinin (WGA; blue). DNA was visualized by DAPI (red). In this experiment 8% (+/- 4%) of the DNA particles show a CPn0809 signal. Pictures were acquired via confocal microscopy. Two different visual fields are shown. Scale bar, 5 μm.

In order to gain further insight into the subcellular localization of CPn0809, infectious EBs were exposed to ionic and non-ionic detergents in aqueous buffer (Fig 5A). The PBS buffer extracted neither intracellular EF-Tu nor the surface-located MOMP, while it leached out some GroEL1 and CPn0809. However, neither 1% Triton X-100 nor 2% Sarkosyl extraction solubilized all of CPn0809, indicating that about half of the protein remained in the Sarkosyl-insoluble chlamydia outer membrane complex (cOMC) fraction. Thus, a fraction of CPn0809 is easily accessible, while the rest is membrane associated.

In order to learn more about CPn0809 function, we next analyzed the localization of CPn0809 at different time points by confocal microscopy. At 48 hpi and 80 hpi CPn0809 was found to localize exclusively within the inclusion, as defined by the IncA signal that delineates the boundary of the inclusion membrane (Fig 5B). Notably, punctate CPn0809 signals are sometimes detected in gaps within the IncA signal. However, no CPn0809 signals were detected beyond the limits of the IncA-labeled inclusion membrane.

The abundance of EB-associated signals in inclusions at 80 hpi suggested that CPn0809 might be located on infectious EBs. Therefore, we studied the localization of CPn0809 at the earliest phase of infection under conditions that permit adhesion but not invasion. Quantification revealed that at this time point (0 min pi) 8% +/- 4% of the CPn0809 signals co-localized with the bacterial DNA signals and with the WGA-stained host-cell membrane (Fig 5C). No CPn0809 signals could be detected within the host-cell cytosol. The relatively low percentage of CPn0809-positive DAPI signals at time point 0 very likely reflects the asynchronous nature of the infection.

**Fig 6:**
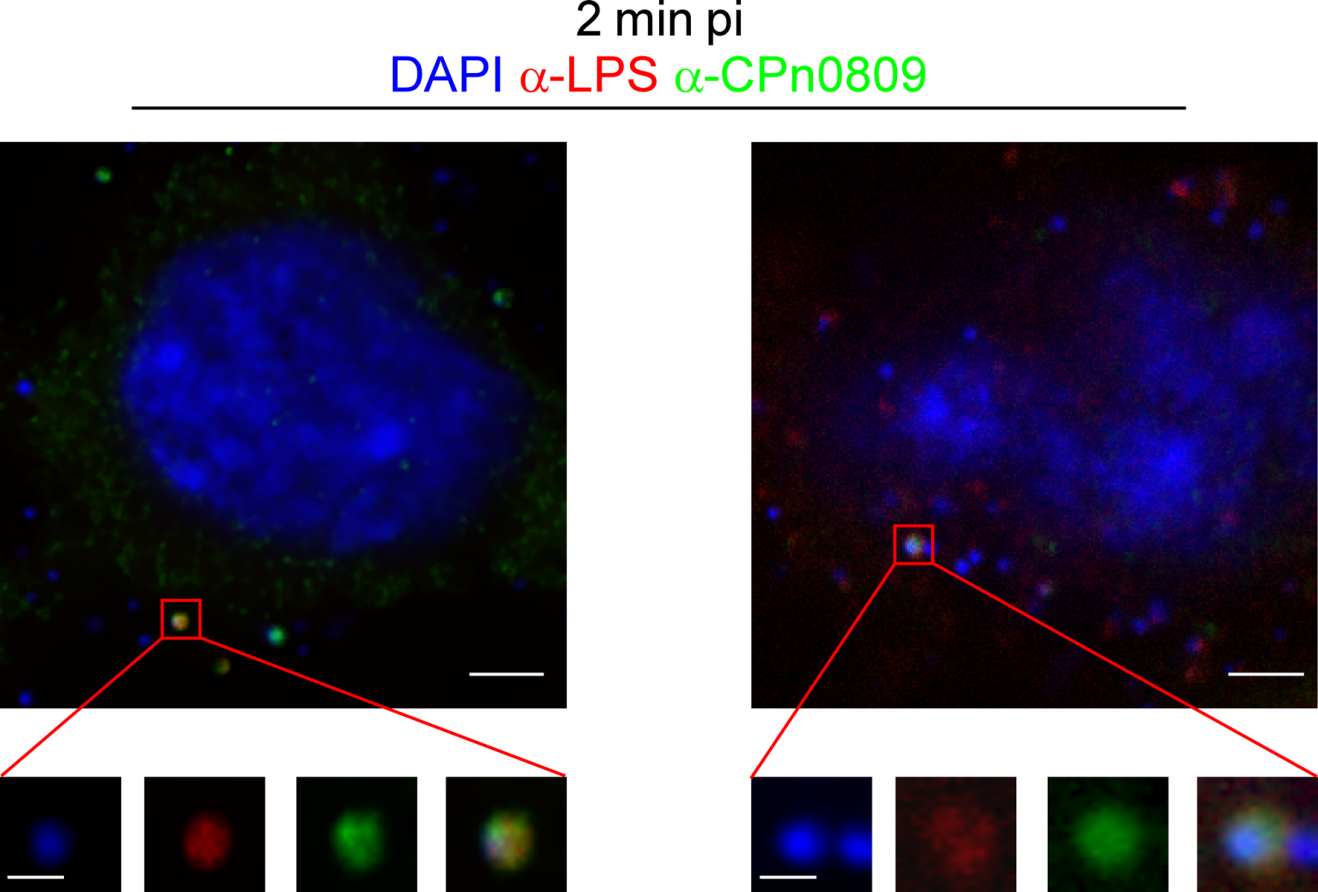
CPn0809 co-localizes with chlamydial LPS at very early stages of infection. HEp-2 cells were infected as described in Fig 5C, fixed with cold methanol at 2 min post infection and stained with an anti-CPn0809 antibody and an Alexa-488-conjugated secondary antibody (green) and an anti-LPS antibody and an Alexa-594-conjugated secondary antibody (red). The demarcated sections are shown enlarged in the bottom row. In this experiment 6% (+/- 2%) of the DNA signals showed a CPn0809 signal. Scale bar, 3 μm and 0.5 μm (in the enlarged sections).

To further investigate CPn0809 localization during adhesion/internalization we shifted cells to 37°C to allow secretion of effectors and internalization of the EBs, and fixed cells with methanol 2 min after media exchange (Fig 6). The co-localization of CPn0809 and LPS at 2 min pi is shown in the enlarged views of CPn0809- and LPS-positive particles marked in the overview panels. At this time point 6% +/- 2% of the DAPI signals co-localized with CPn0809 signals, again reflecting the asynchronous nature of the early infection. Thus it can be concluded that CPn0809 co-localizes with chlamydial EBs during adhesion and internalization.

### Y2H analysis shows translocon-specific interaction of CPn0809 via its N-terminal domain

Our bioinformatic data suggested that CPn0809 might be the major hydrophobic translocator protein of the *C*. *pneumoniae* LcrH_1 T3SS. To find out if CPn0809 shows functional features shared by T3SS translocon proteins from other species, such as oligomerization or chaperone binding, we performed yeast two-hybrid analyses. The hydrophobic translocon proteins of other pathogenic bacteria exhibit self-interaction and possess binding domains for interaction with the corresponding T3S chaperone in their N-terminal segments [18, 40–42]. For this reason, and because the full-length CPn0809 protein was found to be toxic to yeast cells (not shown), we performed these interaction experiments exclusively with the N-terminal half of CPn0809 (aa 1–253), which harbors two predicted coiled-coil domains.

**Fig 7:**
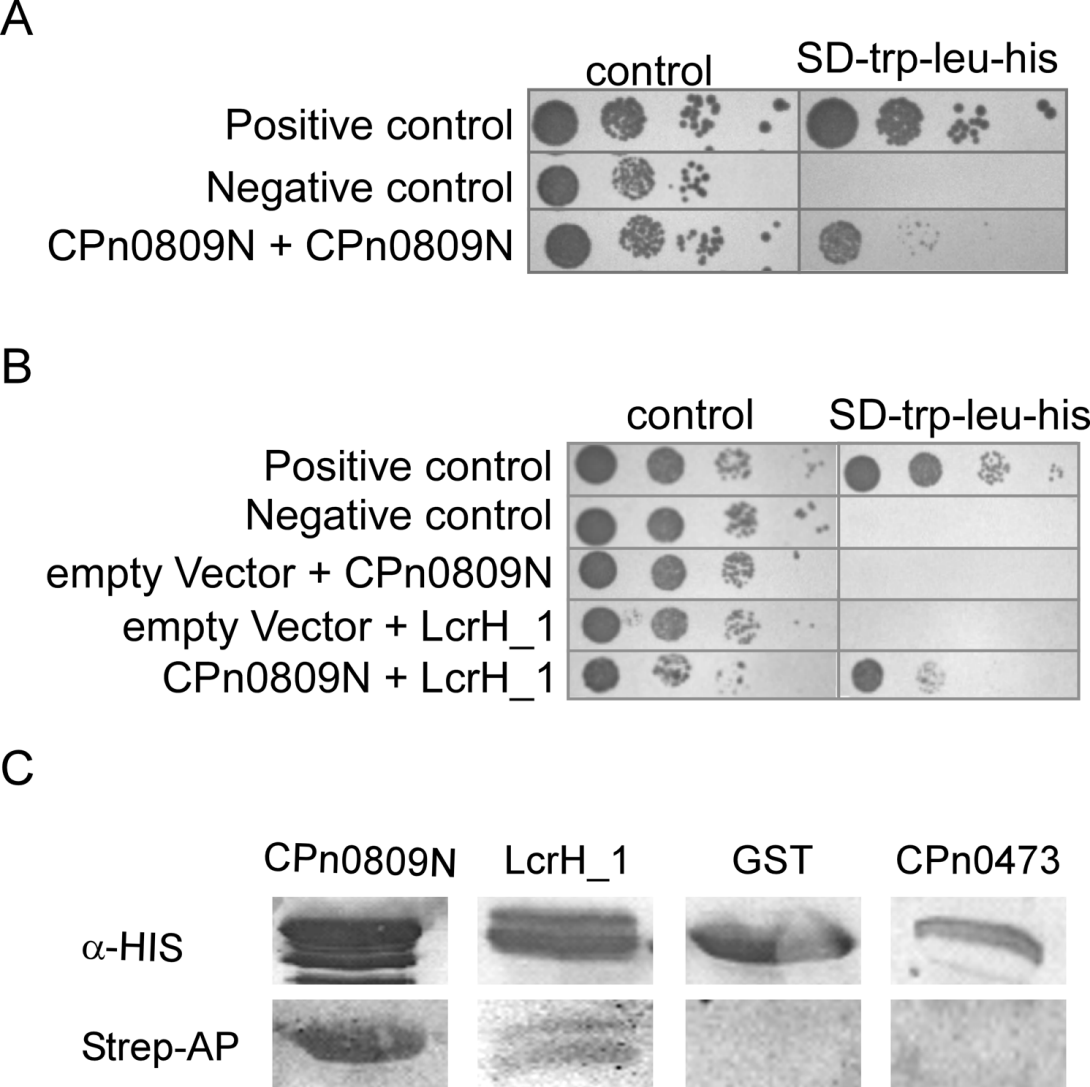
CPn0809 interacts via its N-terminal segment both with itself and with LcrH_1. **A and B.** A CPn0809N-coding (aa 1–253) DNA fragment was fused with the sequence coding for the *GAL4*-DNA-binding domain in the bait plasmid pGBKT7. LcrH_1 and CPn0809N coding sequences, respectively, were likewise fused to the *GAL4* activation domain in the prey plasmid pGADT7. Yeast cells transformed with bait and prey plasmids as indicated were grown in selective liquid media. As a positive control pGBKT7 expressing p53 (pGBKT7-53) and pGADT7 expressing the large T-antigen of SV40 (pGADT7-T), and as a negative control pGBKT7 coding for lamin C (pGBKT7-Lam) and pGADT7-T, were co-transformed. About 10^4^, 10^3^, 10^2^ and 10 yeast cells were spotted on different selection media (control: SD-trp-leu; yeast two-hybrid reporter activation: SD-trp-leu-his) in a serial dilution patch test. Plates were incubated for 48 h at 30°C. Neither CPn0809 nor LcrH_1 showed auto-activation. **C.** *E*. *coli* cells expressing either His_6_-CPn0809N-GST, His_6_-LcrH_1, His_6_-GST or CPn0473-His_10_ prey protein were lysed, and 50 μg of each soluble protein was fractionated by SDS PAGE, blotted onto a PVDF membrane and renatured by stepwise exposure to decreasing concentrations of guanidine-HCl. The prey proteins were visualized using His-specific primary antibodies and an alkaline phosphatase-conjugated secondary antibody (top). The renatured membrane was incubated with biotinylated His_6_-CPn0809N (bait protein). The interaction of bait and prey proteins was then analyzed using streptavidin-conjugated alkaline phosphatase (bottom) (n = 2). M = protein size marker.

Yeast strain AH109 co-expressing CPn0809N-BD and CPn0809N-AD grew on plasmid selective medium as well as low-stringency selective medium (Fig 7A). Their interaction activates expression of the Y2H reporter gene, albeit not as strongly as in the positive control (pGBKT7-p53 and pGADT7-T). We next tested whether CPn0809 can interact with its chaperone LcrH_1 (Fig 7B). Again, yeast cells co-expressing both proteins grew on selective media, indicating activation of the Y2H reporter gene by interaction of the proteins. Next, we wanted to confirm the genetic interaction data by means of a biochemical assay. Using Far Western experiments, we were able to confirm the observed interactions of recombinant CPn0809 with itself and with recombinant LcrH_1 via its N-terminal segment, but not with recombinant GST or the recombinant *C*. *pneumoniae* protein CPn0473, a novel chlamydial cell-surface protein (unpublished) (Fig 7C). Taken together, these data thus showed that CPn0809 is able to interact with the putative T3S-related class II chaperone LcrH_1 and with itself via its N-terminal domain.

### Expression of recombinant CPn0809 is toxic for *E*. *coli*

**Fig 8:**
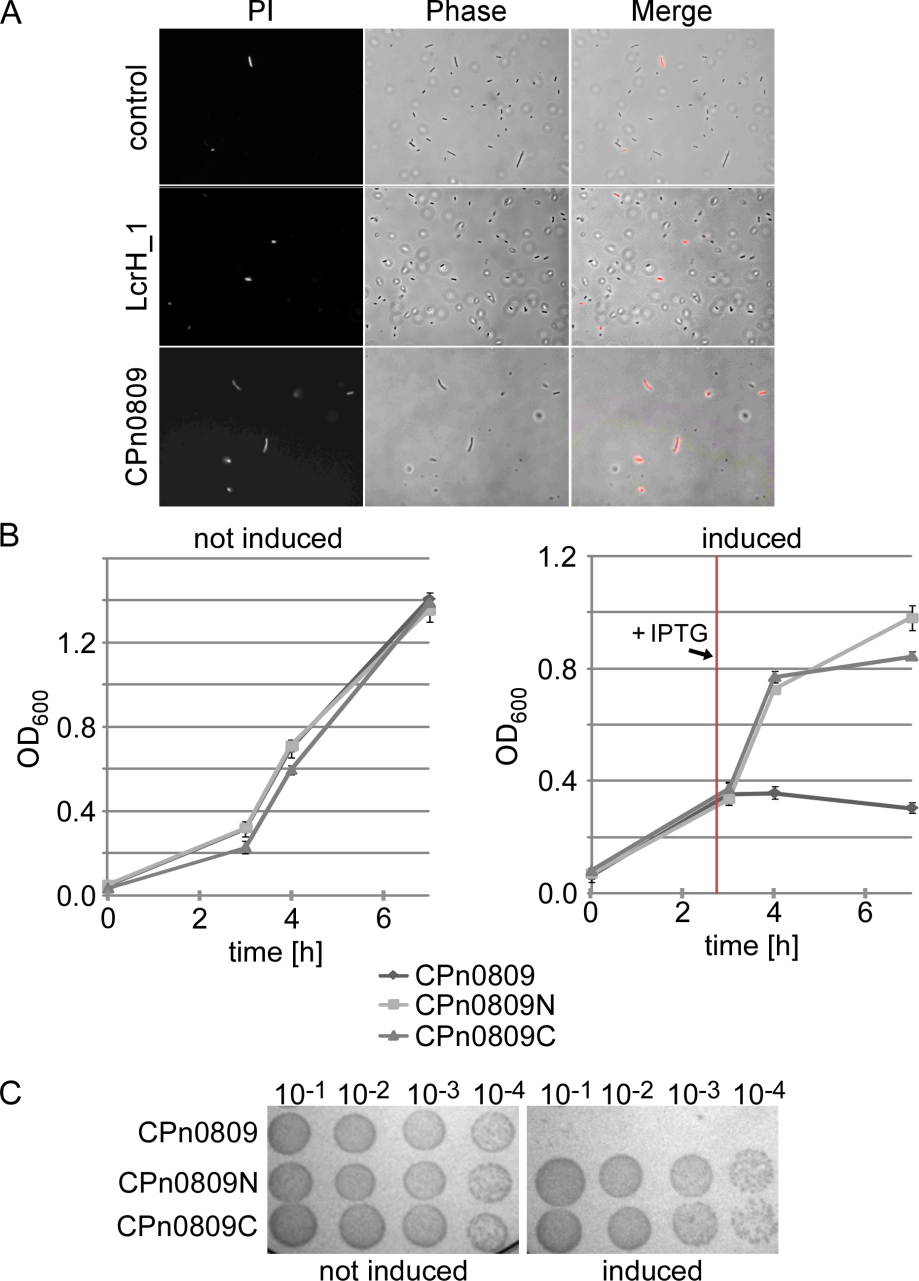
Expression of CPn0809 is toxic to *E*. *coli*. **A.** Vital staining with propidium iodide (PI) of *E*. *coli* cells harboring different plasmid constructs as indicated. Expression of His_6_-CPn0809 and as controls His_6_-LcrH_1 and the empty vector were induced for 2 h. 1 OD_600_ *E*. *coli* cells were harvested and stained with PI as described in the methods and were immediately analyzed by fluorescence microscopy. **B.** Growth curves of *E*. *coli* harboring different plasmid constructs under inducing or non-inducing conditions. Expression of His_6_-CPn0809, His_6_-CPn0809N (aa 1–253) and His_6_-CPn0809C-GST (aa 406–488) was induced by the addition of IPTG (+IPTG, red mark). Experiments were performed in selective liquid media containing 1% glucose. The mean of three independent replicates is shown. **C.** The toxic effect of the expression of CPn0809 in *E*. *coli* was confirmed in serial dilution patch tests of *E*. *coli* transformants shown in B grown under induced or non-induced conditions. Liquid cultures were grown for equal time periods and expression of proteins was induced by the addition of IPTG for 1 h. 10 μl samples were taken and diluted ranging from 10^−1^ to 10^−4^. Dilutions were spotted onto solid LB-media with 1% glucose (repressing condition) and incubated overnight at 37°C.

When we cloned a *his*_*6*_-*cpn0809* full-length construct to produce recombinant protein in the heterologous host *E*. *coli* for further biochemical studies, we found that the transformed strain could be cultured only if we repressed the basal activity of the *lac* promoter by adding glucose (1%) to the medium. After protein expression was induced, the optical density of the cultures, as well as the number of cells per milliliter, showed no further increase over time, in contrast to cells expressing the N-terminal segment of CPn0809 or the empty vector (Fig 8B, S2 Fig) and only small amounts of protein could be detected by immunoblot analysis. These data suggested that expression of CPn0809 in *E*. *coli* might be harmful to the cells, as in the case of the *Yersinia* major translocator YopB [43].

To determine whether CPn0809 has a growth-inhibiting effect, we performed viability assays using propidium iodide (PI), to which living cells are impermeable. Intercalation of PI into DNA in dead cells, on the other hand, gives rise to red fluorescence. *E*. *coli* was transformed with plasmids expressing either His_6_-CPn0809, His_6_-LcrH_1, or the empty vector control and the cultures were analyzed after incubation for 2 h under inducing conditions (Fig 8A). Bacteria carrying the empty vector control or the His_6_-LcrH_1 expressing plasmid showed viable normal-sized *E*. *coli* cells with only a few cells appearing red indicating cell death. In contrast, staining with PI revealed that most cells carrying the His_6_-CPn0809 expression plasmid were inviable, indicating that expression of CPn0809 is indeed toxic to *E*. *coli*.

For the CPn0809 homolog YopB from *Yersinia* it was proposed that the toxic effect of expression in *E*. *coli* was due to the presence of predicted hydrophobic domains (HD) within protein [43]. Thus, we generated N-terminal (aa 1–253) and C-terminal (aa 406–488) CPn0809 expression clones excluding the HD (see Fig 2) and tested them in comparison to the full-length clone in a time-course experiment. Under non-inducing conditions, the growth rates of the three expression cultures were almost identical (Fig 8B). Upon induction of protein expression, cultures expressing either the N-terminal or the C-terminal part of CPn0809 continued to grow, albeit somewhat more slowly, suggesting a possible cytotoxic effect on *E*. *coli* growth. However, cell counts performed 2 h post induction of the CPn0809N-expressing culture were indistinguishable from those of cultures inoculated with cells carrying the empty vector control pKM32, thus excluding a cytotoxic effect (S2 Fig). In contrast, cultures expressing the full-length CPn0809 protein stopped growing, indicating that expression of the chlamydial protein, unlike that of CPn0809N- and CPn0809C, results in complete inhibition of the growth of *E*. *coli* cells (Fig 8B, S2 Fig). To find out whether CPn0809 expression causes growth arrest or actually kills the cells, we spotted cells from cultures after 1 h of protein expression, and cells from non-induced control cultures, in different dilutions on solid medium containing 1% glucose to completely repress protein expression, in order to determine the numbers of colony-forming units left after 1 h of Cpn0809 expression (Fig 8C). Cells from the three non-induced cultures showed an identical regular growth pattern on plates. In contrast, *E*. *coli* cells that had been induced to express full-length CPn0809 showed no growth at all when patched on the non-inducing medium, while growth was not restricted in samples harboring the CPn0809N- and CPn0809C-expressing cells.

Taken together, our findings show a massive cytotoxic effect of full-length CPn0809 on *E*. *coli* cells. CPn0809N and CPn0809C do not show a toxic effect when expressed in *E*. *coli* cells leading us to the assumption that the toxicity of CPn0809 is indeed dependent on the predicted hydrophobic domains and/or on the presence of N- and C-terminus.

## Discussion

The Type III secretion system constitutes a highly sophisticated nanomachine with which pathogenic bacteria deliver effector proteins directly into the cytosol of targeted eukaryotic cells. T3S systems thus play an essential role in enabling intracellular parasites to invade, and replicate in, host cells. The translocon is a protein complex composed of three proteins at the tip of the T3SS needle that forms a pore in the plasma membrane of the target cell. The translocon proteins of *Chlamydia* are poorly understood, although the two predicted translocon operons LcrH_1/2 were identified more than a decade ago [15]. On the other hand, several translocons of T3SS have been investigated extensively in other bacteria and show diversity in composition and function, indicating that each translocon is adapted to the specific niche colonized by the pathogen [12, 13].

In this study, we characterized the putative translocon protein CPn0809, which is encoded within the LcrH_1 operon of *C*. *pneumoniae* [16]. Our bioinformatic analysis indicate that common structural features of major translocon proteins, such as hydrophobic domains surrounded by coiled-coil structures, are present in CPn0809. CPn0809 contains three predicted transmembrane domains (aa 254–275, aa 280–302 and aa 383–405) and three coiled-coil domains (aa 121–140, aa 210–247 and aa 411–436) and their numbers and relative positions within the protein are reminiscent of the domain structure of the major translocators of other bacteria (Fig 2). Direct comparison of the full-length sequences of CPn0809 and other major translocator proteins using BLASTp identified moderate homology to the major translocators SipB (identity 29%; homology 52% with a coverage of 38%) encoded in pathogenicity island I of *Salmonella enterica* and IpaB (identity 30%; homology 49% with a coverage of 38%) of *Shigella flexneri*. Additionally, an ‘SseC-like family’ domain was identified in the C-terminal portion of CPn0809 (aa 203–487). SseC domains are found in the major hydrophobic translocator proteins of all bacterial T3SS considered in this study, while most minor hydrophobic translocators lack them (see Fig 2). However, an ‘SseC-like family’ domain also occurs in the minor hydrophobic translocator specified by the *C*. *pneumoniae* LcrH_1 operon. Moreover, neither of the hydrophobic translocators encoded by the LcrH_2 operon possesses such a domain, implying that, in this respect, *Chlamydiae* differ from other bacteria. Never the less, both putative translocator proteins encoded by the LcrH_2 operon display the other prominent features of hydrophobic translocon proteins, e.g. hydrophobic domains and coiled-coil domains (not shown). Thus, the proteins encoded by the LcrH_1 and LcrH_2 operons might possibly help secreting different classes of effectors during the infection cycle.

By monitoring the expression of CPn0809 throughout the *C*. *pneumoniae* life cycle by means of immunoblot analyses, we found that CPn0809 is readily detectable in the very early stages of infection (0–6 hpi) when the bacteria are still in the EB state. Subcellular localization studies revealed that CPn0809 can be detected on, and is associated with EBs from 0 min pi until 1 hpi. However, only a fraction of DNA signals are associated with a CPn0809 signal and this varies depending on the batch of purified EBs. It is well known that the chlamydial infection cycle does not proceed synchronously, even after prolonged growth [5, 44]. This is particularly relevant for *C*. *pneumoniae*, for which it was recently shown by cryo-EM that at 72 hpi, less than 30% of the bacteria within the inclusion are EBs [44]. Thus the majority of chlamydial particles within the chlamydia pool used for infection experiments is non-infectious. Moreover, even the initial infection step does not proceed in a synchronous manner. In order to maximize synchronization, a short, gentle centrifugation step after EB addition is performed. However, this is only partially successful. Furthermore, the EB purification routine involves different experimental steps, and it has been reported that these methods can deleteriously affect components of the T3SS and alter the physiological form of the bacteria by mechanical stress [45]. This point is particularly relevant here, as we believe CPn0809 to be a translocator of the T3SS needle tip. Thus, in conclusion, the fact that the number of infectious *C*. *pneumoniae* particles in the EB pools is low, and the asynchronicity of the infection process, can together account for the small fraction of chlamydial particles with associated CPn0809 signals observed at every time point analyzed.

During the differentiation of EBs to RBs and the subsequent replication of the latter, CPn0809 was no longer detectable microscopically. The signal reappeared at 48 hpi, when cell division ceases and the asynchronous re-differentiation of RBs into EBs begins. Thus, immunoblot analysis and immunofluorescence microscopy showed that CPn0809 is a late expressed protein. This is consistent with mRNA transcription data, which suggested that CPn0809 is a “tardily” expressed protein that is stored in the EBs, presumably because its function is required in the earliest stages of the subsequent infection process [16, 17].

Subcellular localization studies further revealed that, from 48 hpi on, punctate CPn0809 signals appear within the inclusion, and increase in number until the end of the developmental cycle. At no time point examined was CPn0809 secreted into the cytosol of the host cell. Thus, our study offers no support for the contention that CPn0809 is localized in the inclusion *and* in the surrounding host cell cytosol [19]. Interestingly, the minor hydrophobic translocator CPn0808 of *C*. *pneumoniae* TW183 also localizes within the inclusion and is not secreted into the inclusion membrane or the host-cell cytosol [46]. Additionally, the CPn0808 expression pattern resembles that of CPn0809 [46]. Interestingly, the localization pattern observed for CPn0809 differs from that reported for its *C*. *trachomatis* homolog CopB, which was found to be associated with the inclusion membrane at 20 hpi [47]. These differences may point to species-specific functional differentiation of CPn0809/CopB.

Interestingly, our analyses show that CPn0809 is associated with infectious EBs and that a fraction of it can be readily detached from infectious EBs (Fig 5A). Because the hydrophobic translocators are in most cases not secreted until contact is established with the potential target cell [12], this leads to the suggestion that some CPn0809 might be preloaded into the T3S needle, and ready for secretion upon detection of host-cell contact within the first minutes of infection. In addition, CPn0809 signals are associated with the chlamydial LPS during adhesion and internalization. Thus CPn0809 could participate in translocation pore formation during attachment and might be important for the secretion of early effector proteins such as TARP, which is an important prerequisite for EB uptake, as it re-organizes the actin cytoskeleton.

For its predicted function as a translocation-pore protein it is crucial that CPn0809 be able to form homo- and/or hetero-oligomers. Importantly, we found that the N-terminal part of CPn0809 (aa 1–253) alone mediates both self-interaction and interaction with its proposed chaperone LcrH_1 *in vivo* and *in vitro*, as is true of well characterized major translocators in other bacteria [40–43, 48]. Interaction with their chaperone is an essential characteristic of both hydrophobic translocators. Binding of the chaperone keeps the translocators unfolded, protects them from degradation and prevents premature oligomerization, which is toxic for bacterial cells [43, 48, 49]. Following secretion, the translocon proteins form oligomers to build up the translocon within the plasma membrane of the targeted cell. Other translocon proteins are also capable of assembling into homo-oligomers [50].

Finally, we analyzed the growth-inhibiting effect of CPn0809 in *E*. *coli* in more detail, and found that while expression of the CPn0809 full-length protein leads to cell death, synthesis of CPn0809N or CPn0809C, both of which lack the hydrophobic domains, does not. This implies that the transmembrane domains and/or the N- and C-terminal domains together are responsible for the growth inhibition phenotype observed here. For other translocon proteins it has been shown that heterologous expression in *E*. *coli* leads to cell lysis, which is also dependent on the presence of the transmembrane domains [43]. Transmembrane domains play a crucial role in anchoring proteins into membranes. The CPn0809 fragments used here lack predicted transmembrane domains, are probably not inserted into membranes and are thus unable to form pores. Induction of heterologous expression of other chlamydial proteins that are capable of pore formation, such as MOMP and Pom proteins, also provokes a drop in optical density in *E*. *coli* expression culture, implying cell lysis [51, 52]. One could speculate that, in our case, the amount of CPn0809 protein produced upon induction of expression is too low to induce cell lysis, but results in cell death.

Recently, the Mahony group demonstrated that the minor translocator of *C*. *pneumoniae*, CPn0808 (now re-named CopD), displays characteristics which are compatible with the assumption that it acts as one of the *C*. *pneumoniae* translocators [18]. They identified the CopD chaperone interaction motif PxLxxP (aa 120–125), which is essential for binding of LcrH_1. A similar motif was found in CPn0809 (aa 166–171) [18], which lies within the N-terminal domain of CPn0809 examined in our yeast two-hybrid analysis. Furthermore, it was shown that CopD also forms oligomers in solution. In future studies, it would be interesting to analyze the interaction between *C*. *pneumoniae* CPn0809 and CopD in more detail.

Interestingly, CopD is essential for infection, as it has been reported that treatment of EBs with an anti-CopD peptide antibody prior to infection reduces infection by up to 98% [18]. This strong neutralization phenotype suggests the presence of CopD at the T3SS needle tip of infectious EBs, a location that is uncommon for hydrophobic translocators prior to host-cell contact [12]. Thus far, only one example has been reported of a hydrophobic translocator that is already present at the tip of the T3SS needle prior to contact with the host cell: The *Shigella* IpaB protein, which together with the hydrophilic IpaD forms the tip complex and is assumed to be involved in host cell sensing [13]. The accessibility of CopD to antibodies and their subsequent inhibitory effect on the infection seem to be specific for this chlamydial protein, as CPn0809 could not be detected on the EB surface during the first minutes of infection, in the absence of permeabilization. However, we cannot exclude the possibility that our antibody, which was raised against the N-terminal 253 amino acids, detects an epitope that is not exposed prior to insertion into the host cell membrane and is therefore not accessible on purified EBs.

In summary, in this report we show that CPn0809 exhibits key features of T3SS translocator proteins. Our data provide strong evidence that support the previously proposed function of CPn0809 as a translocon protein of the T3S apparatus of *Chlamydia pneumoniae*. Consequently, we recommend that CPn0809 be renamed CopB by analogy to the major translocator protein of *Yersinia* YopB, as initially proposed [53].

## Supporting Information

S1 Fig Anti-CPn0809 antibody specificity test. Green fluorescent beads (1 x 10^9^, ø 1.1 μm, Polysciences) were coated either with recombinant GST-CPn0809N-His_6_ (aa 1–253) or with BSA as negative control, and then incubated for 1 h at 4°C with the rabbit anti-CPn0809 antibody in coupling buffer. Subsequently beads were pelleted by centrifugation and the supernatant was used to stain HEp-2 cells 48 h post infection with *C*. *pneumoniae* (upper panel). Binding of the anti-CPn0809 antibody to the beads was visualized by staining the beads with a secondary anti-rabbit Alexa-594 antibody (lower panel). Analyses were performed by fluorescence microscopy.
(TIF)

S2 Fig Growth curves of *E*. *coli* cells harboring different plasmid constructs after induction with IPTG. Expression of His_6_-CPn0809, His_6_-CPn0809N (aa 1–253) and the empty vector (pKM32) was induced for 2 h by the addition of IPTG. Experiments were performed in selective liquid media containing 1% glucose. Cell numbers were determined by measuring absorbance at 600 nm (A) and by cell counting (B). The mean of three independent replicates is shown.
(TIF)

## Abbreviations

CC: coiled-coil protein domain
cOMC: chlamydia outer membrane complex
EB: Elementary Body
HD: hydrophobic protein domain
hpi: hours post infection
MOI: multiplicity of infection
MOMP: major outer membrane protein
PI: post infection
RB: Reticulate Body
T3SS: Type III secretion system
WGA: Wheat germ agglutinin
