# Supplementary figures and images for: The Type III Secretion System-Related CPn0809 from Chlamydia pneumoniae
Source: PLoS One. 2016 Feb 19;11(2):e0148509. doi: 10.1371/journal.pone.0148509 (PMC4760673; doi:10.1371/journal.pone.0148509)

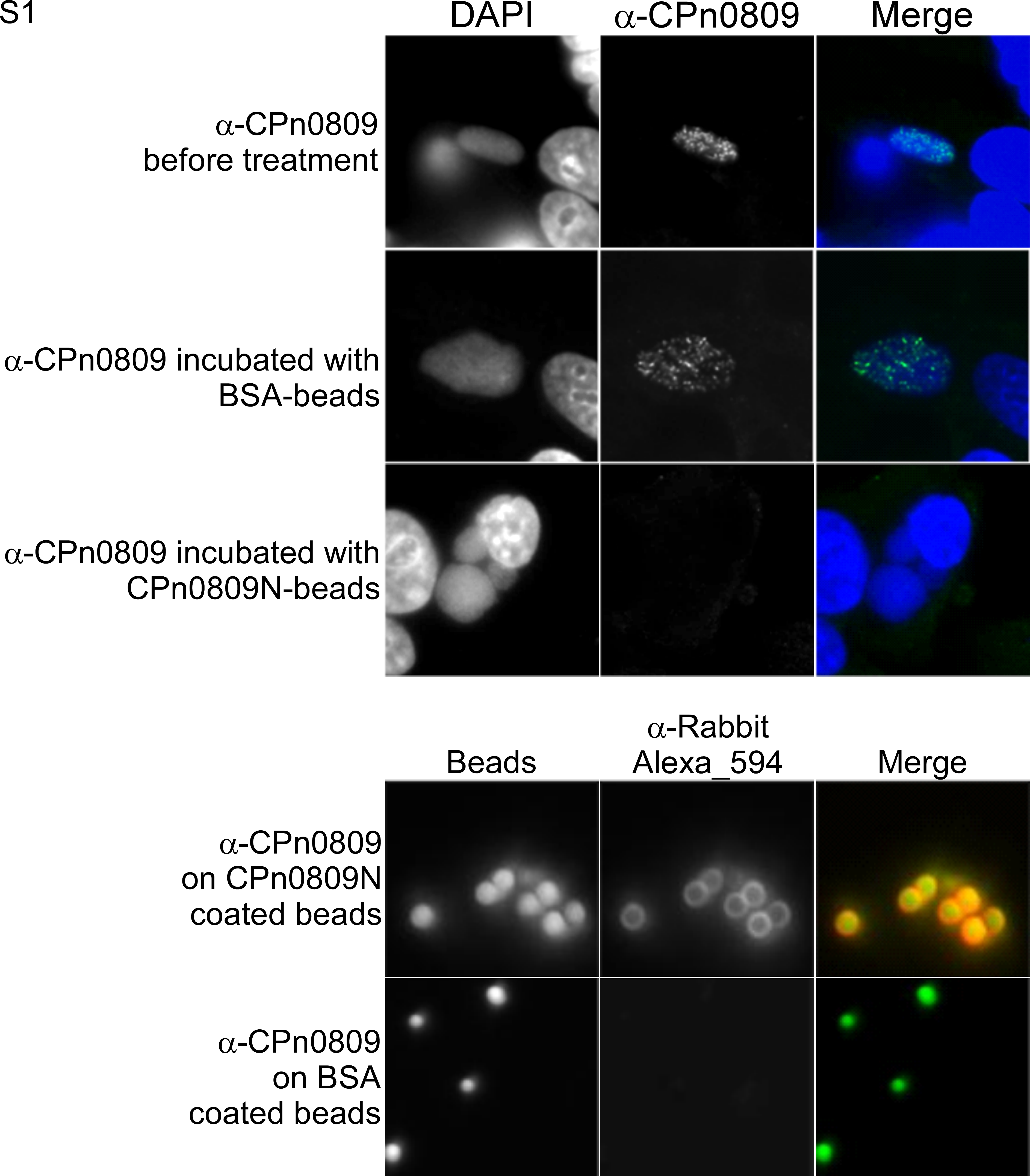

Supplement: S1 Fig — Green fluorescent beads (1 x 10^9^, ø 1.1 μm, Polysciences) were coated either with recombinant GST-CPn0809N-His6 (aa 1–253) or with BSA as negative control, and then incubated for 1 h at 4°C with the rabbit anti-CPn0809 antibody in coupling buffer. Subsequently beads were pelleted by centrifugation and the supernatant was used to stain HEp-2 cells 48 h post infection with C. pneumoniae (upper panel). Binding of the anti-CPn0809 antibody to the beads was visualized by staining the beads with a secondary anti-rabbit Alexa-594 antibody (lower panel). Analyses were performed by fluorescence microscopy. (TIF) [file pone.0148509.s001.tif]

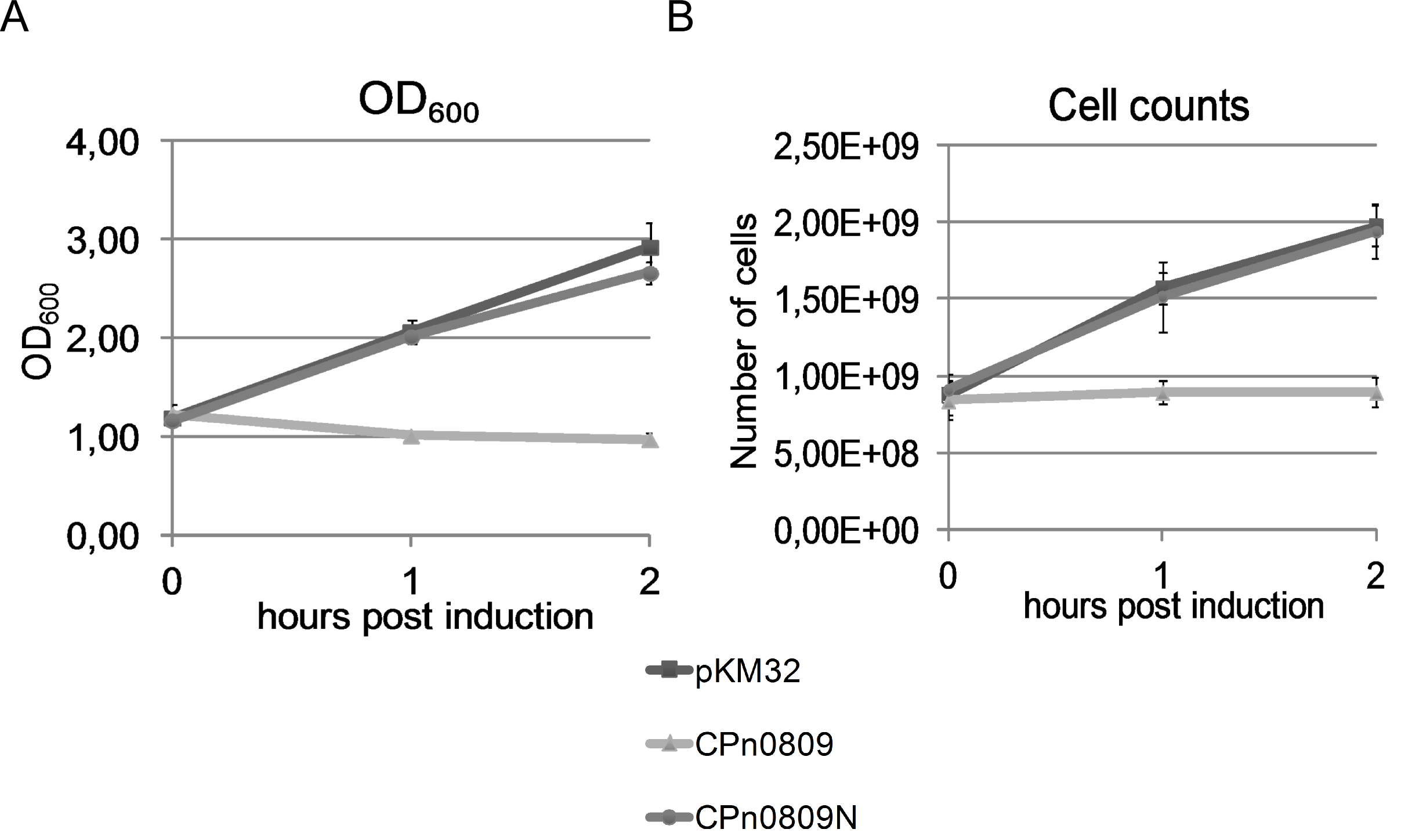

Supplement: S2 Fig — Expression of His6-CPn0809, His6-CPn0809N (aa 1–253) and the empty vector (pKM32) was induced for 2 h by the addition of IPTG. Experiments were performed in selective liquid media containing 1% glucose. Cell numbers were determined by measuring absorbance at 600 nm (A) and by cell counting (B). The mean of three independent replicates is shown. (TIF) [file pone.0148509.s002.tif]
